# Concentration Determination of >200 Proteins in Dried Blood Spots for Biomarker Discovery and Validation*[Fn FN2]

**DOI:** 10.1074/mcp.TIR119.001820

**Published:** 2020-01-01

**Authors:** Azad Eshghi, Adam J. Pistawka, Jun Liu, Michael Chen, Nicholas J. T. Sinclair, Darryl B. Hardie, Monica Elliott, Lei Chen, Rachael Newman, Yassene Mohammed, Christoph H. Borchers

**Affiliations:** ‡ University of Victoria - Genome BC Proteomics Centre, University of Victoria, Victoria, British Columbia V8Z 7X8, Canada; § Department of Pathology and Laboratory Medicine, University of British Columbia, Vancouver, British Columbia V6T 2B5, Canada; ¶ Island Medical Program, Department of Pathology and Laboratory Medicine, University of British Columbia, Vancouver, British Columbia V6T 2B5, Canada; ‖ Center for Proteomics and Metabolomics, Leiden University Medical Center, Albinusdreef 2, 2333 ZA Leiden, The Netherlands; ** Department of Biochemistry and Microbiology, University of Victoria, Victoria, British Columbia V8P 5C2, Canada; ‡‡ Segal Cancer Proteomics Centre, Lady Davis Institute, Jewish General Hospital, McGill University, Montreal, Quebec H3T 1E2, Canada; §§ Gerald Bronfman Department of Oncology, Jewish General Hospital, McGill University, Montreal, Quebec H3T 1E2, Canada; ¶¶ Department of Data Intensive Science and Engineering, Skolkovo Institute of Science and Technology, Skolkovo Innovation Center, Nobel St., Moscow143026, Russia

**Keywords:** Multiple reaction monitoring, quantification, blood, biomarker: prognostic, biomarker: diagnostic, biofluids, HPLC, high throughput screening, mass spectrometry, omics, dried blood spot

## Abstract

The use of protein biomarkers as surrogates for clinical endpoints requires extensive multilevel validation including development of robust and sensitive assays for precise measurement of protein concentration. Multiple reaction monitoring (MRM) is a well-established mass-spectrometric method that can be used for reproducible protein-concentration measurements in biological specimens collected via microsampling. The dried blood spot (DBS) microsampling technique can be performed non-invasively without the expertise of a phlebotomist, and can enhance analyte stability which facilitate the application of this technique in retrospective studies while providing lower storage and shipping costs, because cold-chain logistics can be eliminated. Thus, precise, sensitive, and multiplexed methods for measuring protein concentrations in DBSs can be used for *de novo* biomarker discovery and for biomarker quantification or verification experiments. To achieve this goal, MRM assays were developed for multiplexed concentration measurement of proteins in DBSs.

The lower limit of quantification (LLOQ) was found to have a median total coefficient of variation (CV) of 18% for 245 proteins, whereas the median LLOQ was 5 fmol of peptide injected on column, and the median inter-day CV over 4 days for measuring endogenous protein concentration was 8%. The majority (88%) of the assays displayed parallelism, whereas the peptide standards remained stable throughout the assay workflow and after exposure to multiple freeze-thaw cycles. For 190 proteins, the measured protein concentrations remained stable in DBS stored at ambient laboratory temperature for up to 2 months. Finally, the developed assays were used to measure the concentration ranges for 200 proteins in twenty same sex, same race and age matched individuals.

## Abstract (precis)

Multiple reaction monitoring with isotope dilution assays were developed for quantification of 200 proteins in dried blood spots, with median inter-day CV of 8%. Developed assays were used to measure the concentration ranges of these 200 proteins in twenty same sex, same race and age matched individuals. These, precise, sensitive, and multiplexed assays can be applied for *de novo* biomarker discovery and for biomarker quantification or verification experiments.

## Graphical Abstract

**Figure:**
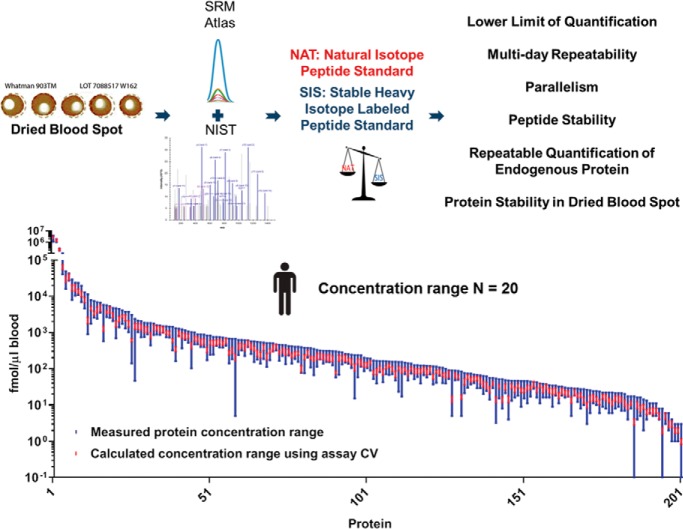

**Highlights**

- Repeatable quantification of 200 proteins in dried blood spots.
- Determined lower limit of quantification, repeatability, parallelism and stability.
- Protein stability in DBS stored at ambient temperatures for up to 2 months.
- Concentration ranges for 200 proteins in 20 healthy individuals.

Blood is the most used biofluid for clinical tests and for research purposes, as it can often indicate the health status of an individual. Although the most common method of blood collection is venipuncture, microsampling methods, requiring <100 μl of blood, such as capillary blood sampling, are gaining acceptance (1). The dried blood spot (DBS)^1^ microsampling technique has already been found to be useful for both population-wide screening and in research studies, as indicated by the total number of analytes currently measurably (using a variety of analytical methods and numerous assays), including 1001 small molecules, 677 large molecules (proteins), 309 nucleic acids and 31 elements (2). DBSs are already used in screening programs world-wide for identification of inborn errors of metabolism and other disorders in newborns (3). The advantages of using DBS sampling include non-invasive collection of blood from heal and finger capillary punctures, which can be performed without the presence of a phlebotomist, and the elimination of the cold chain logistics of shipping and/or storage which leads to a significant reduction in cost.

Despite the advantages noted above, DBS sampling poses significant challenges when the downstream application is the determination of endogenous analyte concentrations. The main pre-analytical variable that needs to be accounted for is the variability in the hematocrit (the % red blood cells in the sample—inter-individual (healthy) range of 30–50%) which can affect the concentrations of the analytes (4–13). DBS sampling also poses a unique challenge in bottom-up proteomics because the drying process may induce conformational changes in protein structure (14) which can alter downstream tryptic digestion efficiency (15, 16). Both the recovery and the tryptic digestion efficiency can be compensated for by using analyte-specific correction factors that are determined by quantifying the target analytes in matched blood and DBS samples (17).

The main analytical challenge for the multiplexed measurement of protein concentration in DBSs using LC-MS is the dynamic range of protein abundance which results in reduced instrument sensitivity. Multiple reaction monitoring (MRM) is a targeted mass spectrometry technique which offers high sensitivity (18) and has been applied to measure protein concentration in DBS (15, 19). Furthermore, the Clinical Proteomics Tumor Analysis Consortium (CPTAC) Assay portal - enhances the awareness and recognition of MRM assay reproducibility by facilitating dissemination of highly validated MRM assays to assist scientists and clinicians in their research, including biomarker validation and discovery (20). The guidelines present a three-tiered system (21) in which biomarker discovery falls in Tier 2, requiring a moderate to a high degree of analytical validation, the use of labeled internal standards for every analyte, high specificity, CVs of 20–35% and high repeatability.

Because the eventual application of biomarkers is for use in screening and/or diagnostic assays, proteomic strategies that can measure actual protein concentrations, as opposed to relative amounts, should be preferred for establishing reference concentration ranges and percentiles that in a clinical setting could be consulted for disease diagnosis (22, 23). The aim of our current study was to develop a repeatable method for measuring the concentrations of proteins in DBSs, with enough precision so that the assay complies with Tier 2 guidelines for targeted MS measurements. Our group has previously demonstrated the precise quantification of 97 endogenous proteins in DBS (15). Here, we built on that study by expanding the protein multiplexing capability of the assay to 200 proteins and improved the validation strategy by performing extended repeatability testing at the lower limit of quantification (LLOQ). We also determined the slope of the response curve for each analyte in different biological replicates and performed extensive stability testing of standards and endogenous proteolytic peptides. Correction factors for each analyte were also determined to compensate for pre-analytical variability and to eliminate the need for volumetric spotting. Finally, the precision of the assays was compared with the biological variability for each protein by measuring of protein concentration in a cohort of same-sex, same-race, age-matched individuals.

## EXPERIMENTAL PROCEDURES

### 

#### 

##### Collection of Human Whole Blood

All blood obtained from commercial vendors was collected at FDA licensed facilities, with a standard blood center consent questionnaire informing the donor that the blood sample may be used for research purposes, without revealing the donor's identity. Blood obtained from Victoria General Hospital was collected from donors with informed consent. The University of Victoria IRB ethics protocol number for using human blood samples for the present study is 14–161.

Human whole blood from venipuncture was purchased from Bioreclamation (Westbury, NY). Five female and 4 male healthy, non-fasting individual donors were recruited to each donate 5 ml of blood which was collected intravenous using citrate phosphate dextrose anticoagulant. Blood samples were pooled and shipped cold without freezing and were subsequently used to generate DBS (described below) within 48 h after collection of blood.

Dried blood spots generated using capillary blood were purchased from DxBiosamples (San Diego, CA). The donor cohort was comprised of 20 Caucasian males ages 19–39 (supplemental Table S1). DBS were spotted according to previously described guidelines (24). A donor would rub his hands together for 5–10 s to stimulate blood flow. Subsequently, the ring finger on the left hand was cleaned with an alcohol pad and allowed to fully dry, and this sequence was performed two times. The hand was securely and comfortably positioned below the heart to facilitate blood flow. The ring finger was grasped, and gentle pressure applied by slowly and firmly squeezing the finger while moving the grasp from the palm to fingertip to pull blood toward the fingertip. A micro-lancet, lancet safety 3.0 mm Unistick2 (HemaSource, West Jordan, UT), was used to puncture the participant's finger perpendicular to the fingerprint, care being taken to not puncture the center of the finger or close to the nail bed; rather aiming for the ulnar side of the finger. The first drop of blood was wiped away with a sterile gauze pad, to minimize non-blood fluids that would be released because of puncturing of surrounding dermal tissue. While maintaining the hand below the heart, a capillary blood collection systems (300 μl RAM Scientific Safe-T-Fill™, EDTA coated, Fisher Scientific, Toronto, ON) was used to collect 300 μl of blood. After completion of blood collection, a pipette was used to spot 50 μl of blood per spot filling all five spots, on a Whatman 903 Protein Saver Card (Sigma-Aldrich, Oakville, ON).

Blood spots were dried contact-free at ambient temperature (between 19 °C and 26 °C) for 3–5 h, with minimal exposure to intense light, and were subsequently placed in a re-sealable zipper bag with a humidity sponge with an indicating desiccant (Fisher Scientific, Toronto, ON). Cards were shipped at ambient temperature and analyzed using MRM within 13 days after the spotting of the DBS samples.

##### Pre-analytical Sample Preparation

DBSs were visually examined for uniformity, and if a spot displayed visual discoloration or clusters of solidified material, it was omitted from further analysis. DBS discs (6 mm) were punched out using a pneumatic card punch (Analytical Sales & Services, Inc., Flanders, NJ). Disc punches were performed by punching each spot west, north, east, south, or center (supplemental Fig. S1). All five discs were collected in a single 1.5-ml conical tube and 1250 μl (*i.e.* 250 μl per punch) of 50 mm ammonium bicarbonate - 2% sodium deoxycholate (DOC) solution was added. Solid-to-liquid-phase extraction from the discs was achieved by incubation for 45 min at 37 °C with shaking at 1400 rpm using an Eppendorf Thermomixer R (Fisher Scientific). Samples were centrifuged for 15 s to pellet the discs and particulates, and positive displacement pipette was used to transfer 200 μl to a new conical tube. A 10-μl aliquot of 0.5 m Bond-Breaker TCEP solution (Fisher Scientific) was added, and the solution was incubated at 99 °C for 15 min with shaking at 950 rpm on an Eppendorf Thermomixer R. Samples were centrifuged briefly at 17,000 × *g*, 30 μl of an aqueous 100-mm iodoacetamide solution was added, and the samples were incubated at 37 °C for 15 min in the dark. Using a positive displacement pipette, 50 μl of the sample solution was transferred to a new conical tube, and 60 μl of TPCK-treated trypsin (>180 units per mg protein, 5.5 μg/μl in 50 mm ammonium bicarbonate) (Worthington-Biochemicals, Lakewood, NJ) was added. Sample was incubated in a thermomixer for 9 h at 37 °C with shaking at 750 rpm. Subsequently, 12 μl of 10% formic acid (in water) was added to inactivate the trypsin and to precipitate the DOC. Sample was centrifuged at 21,000 × *g* for 10 min, and 70 μl of the supernatant was transferred to a 300-μl sample injection vial (PP screw vial, 12 × 32 mm, 9 mm thread, from Canadian Life Science, Edmonton, AB). At this step the sample was ready to be spiked with peptide standards prior to LC-MS/MS analysis.

A surrogate matrix was made for formulating the samples used to generate the calibration curves. Unused DBS extracts which had been processed up to the addition of trypsin, were pooled to make a surrogate matrix (SM). To 500 μl of pooled DBS extract, 600 μl of proteinase K (≥30 units/mg protein, 5.5 μg/μl, in 50 mm ammonium bicarbonate) (Sigma-Aldrich) was added and sample was incubated in an Eppendorf Thermomixer R at 37 °C for 9 h with shaking at 750 rpm. To stop the digestion and precipitate the DOC, 120 μl of 10% aqueous formic acid was added to the reaction mixture. The sample was centrifuged for 10 min at 21,000 × *g* to pellet the DOC and any other debris, and the supernatant was transferred to a 5000 MW-cut-off ultra-filtration conical tube (Agilent Technologies, Cedar Creek, TX) and centrifuged 4000 × *g* for 45 min to remove the proteinase K from the surrogate matrix. The flow-through was used for preparing the samples for generating the calibration curve.

##### Protein Identification Using DBS

Proteins were selected for MRM assay development based on their detectability in DBSs, and were identified by performing bottom-up proteomics in-house. After pre-analytical sample preparation (described above) of two replicate DBS samples, the samples were subjected to solid phase extraction (25) and lyophilisation. Samples were solubilized in 210 μl of 3% aqueous acetonitrile in water and used for LC-MS/MS on an Orbitrap Fusion Tribrid coupled to an EASYnLC 1000 HPLC system via a Nanospray Flex NG source (Thermo Fisher Scientific), as previously described (26, 27). The only modification made was to inject 1 μl of each technical replicate, which was equivalent to ∼ 1 μg of total protein. Raw files were created using XCalibur 3.0.63 (Thermo Scientific) software and analyzed with Thermo Proteome Discoverer 2.2.0.388 software suite (Thermo Scientific). The raw data files were processed as previously described (26) with the following modifications: the peak lists were submitted to Mascot 2.4.1 and Sequest servers using the UniProt human database (28) which contained 20,338 protein sequences. The enzyme was set to Trypsin (Full) with max missed cleavage set to 2. The fixed modification was set to carbamidomethyl and variable modifications were set as oxidation (M), deamidation (NQ), and N-terminal methionine loss. Precursor and fragment mass tolerances were set at 8 ppm and 0.8 Da, respectively. Peptide spectral match validation was performed using Percolator setting the maximum Delta Cn to 0.05 and decoy database searches at 0.01 for target FDR (Strict), 0.05 (relaxed) and validation based on q-Value. Proteins were selected for MRM assay development by consulting PeptideTracker (29) for available MRM compatible stable isotope labeled standard (SIS) peptides. Using this resource, 334 SIS peptides, targeting 270 unique proteins (for select proteins multiple unique SIS peptides were available), were selected for MRM assay development.

##### Targeted MRM

Targeted MRM analysis of the final MRM assay panel was performed on an Agilent 6490 triple quadrupole mass spectrometer connected to a 1290 Infinity UHPLC system via a jet stream ESI source (Agilent Technologies). The column compartment was equipped with a Zorbax Eclipse Plus C18 rapid resolution HD 2.1 × 150 mm 1.8-micron column (Agilent Technologies) and an upstream 0.3 μm in-line filter. UHPLC was performed by setting up a 60 min gradients at a flow rate of 0.4 ml/min. The mobile phases were 0.1% formic acid (FA) in water and 0.1% FA in ACN. The gradient was set as follows: 2% ACN, 0 min; 2% ACN, 4 min; 7% ACN, 6 min; 30% ACN, 54 min; 45% ACN, 57 min; 80% ACN, 57.5 min; 80% ACN, 59.5 min; 2% ACN, 60 min. Post-gradient equilibration time was set to 4 min and the stop-time was set to 60 min. The column compartment was kept at 50 °C.

Targeted MS acquisitions were performed using 3-min detection windows, a 970-ms cycle time, and ≥10 ms and ≤ 400 ms dwell times. Three time segments were used for dynamic MRM as follows: at a start time of 0 min, the valve was diverted to waste, at 4 min the valve was diverted to the MS, and at 57.5 min the valve was diverted to waste. A 15-min wash, where 8 μl of 3% aqueous ACN was injected, was included after each DBS sample run to minimize carryover. At the end of each worklist, ten 5-min wash steps were included, injecting 20 μl of 3% aqueous ACN to wash the LC capillary lines from the injection needle onwards, and for these injections, the MS valve was diverted to waste. The Dynamic MRM method for a single transition acquisition is included in supplemental Table S1. The source settings were set as follows; gas temperature 150 °C, gas flow 15 L/min, nebulizer 30 psi, sheath gas temperature 250 °C, sheath gas flow 11 L/min, capillary 3500 V and 3000V (-ve) for positive and negative ion funneling, respectively, nozzle voltage was 300 V and 1500 V for positive and negative ionization, respectively, iFunnel parameters for high pressure RF was 200 V for positive mode and 150 V for Negative mode and low pressure RF was 100 V and 60 V for positive and negative, respectively. System suitability assessment was performed weekly using frozen aliquots of 35 SIS and NAT peptide pairs. Both the HPLC and mass spectrometer performed within the accepted limit of variation which was set at a CV < 20% for measured peak area ratios for all 35 peptide pairs. In other words, the peak area ratios of NAT:SIS for each peptide pair across weekly measurements was less than 20%.

##### Peptide Standard Synthesis

Peptide synthesis was performed as previously described (30). Briefly, SIS peptides were synthesized by incorporating ^13^C/^15^N heavy isotope l-arginine or l-lysine on the C terminus. Peptides were purified using HPLC and the parent mass to charge ratio confirmed using an Ultraflex III-MALDI TOF/TOF Mass Spectrometer (Bruker LTD., Milton, ON). The purity of each peptide was determined using capillary zone electrophoresis on a 7100 Capillary Electrophoresis System (Agilent Technologies), as previously described (30, 31), and the absolute concentration of each peptide was measured in-house using LC/MS-based amino acid analysis (32). Briefly, peptide standards were acid hydrolyzed and the resulting amino acids were dansylated (32) and subjected to HPLC-MS/MS (1290 Infinity HPLC online with 6490 triple Quad MS) (Agilent-Technologies) operated in dynamic MRM mode. Quantification was performed using a ^13^C or ^2^H labeled internal standard for each amino acid (Cambridge isotope Laboratories, Inc., Tewksbury, MA). Synthesis and quantification of the corresponding NAT peptides were performed exactly as indicated above, but without incorporation of heavy-isotope labels. For some of the SIS or NAT peptides, which were not quantified via CZE and AAA, the corresponding NAT or SIS peptides which had been quantified via CZE and AAA were used to accurately measure the concentration of the stock peptide.

##### Selection of Transitions and Initial Assessment of Specificity and Interference

The selection of the MRM transitions was performed using Skyline-daily (33) by cross-referencing tandem mass spectral libraries available through the National Institute of Standards and Technology (NIST) (34). Using these resources, the top five most-intense transitions were selected for collision energy optimization (35) and retention time determination. If a tandem mass spectrum was not available for a target peptide, transitions were characterized empirically by monitoring all y and b-ion fragments using precursor charge states 2 through 5 and corresponding product ions with charge states 1 through 4. SIS peptides were then spiked into the DBS matrix (*i.e.* a tryptic-digested DBS sample) and the combination of retention time and transitions was used to confirm the detectability of each endogenous proteolytic peptide via MRM, and to eliminate transitions prone to interference. As an example, the top five transitions representing the proteolytic peptide (VGEFSGANK), for redox signaling protein thioredoxin (P10599), are shown in (supplemental Fig. S2). The NIST tandem mass spectrum for the same peptide is also included for reference. To maximize assay sensitivity, the collision energy (CE) used to fragment parent peptide ions was optimized as previously described (36). Briefly, the existing linear equation used for predicting the optimal CE for each peptide, in Skyline-daily (33), was first used to create a scheduled MRM method. The scheduled MRM method was then used to create a CE optimization method by setting the step count to 5 and the step size to 1 (5 V on each side of the predicted CE, incremental at 1 V). The CE which produced the highest peak area for each transition was used for downstream methods development. To increase downstream multiplexing, only three transitions were selected for subsequent assay development (supplemental Table S1). The selection criteria used for selecting the three transitions included prioritizing y-ions over b-ions, because the former is more robustly fragmented in low-energy CID used in a triple quadrupole mass spectrometer (37), selecting the highest-intensity product ions to maximize sensitivity, and selecting transitions which displayed consistent peak-area ratios when comparing between the heavy and light versions of a peptide.

### Experimental Design and Statistical Rationale

#### 

##### Response Curve

CPTAC experiment 1 (20) was used as a guide to assess the lower limit of quantification (LLOQ) for each target peptide. Using SIS peptides to spike trypsin digested DBS (DBS matrix), the reverse-curve (26, 27) approach was used to generate an eight-point response curve (in addition to a blank), to determine the LLOQ. The 336 SIS peptides to be tested were used to make working solutions of SIS peptides containing 5000 fmol/μl of peptide in 3% ACN/H_2_O. The working solution of SIS peptide was used to make a dilution series for an eight-point response curve, covering the concentration range from 60 attomoles to 4000 femtomoles of SIS peptide injected on column. At the time the response curve was performed, counterpart NAT peptides were available for use as normalizers for 245 SIS peptides. For those SIS peptides where the corresponding NAT peptide was not available, the endogenous proteolytic analog was used as a normalizer. Additionally, the repeatability was assessed without normalization by measuring the coefficient of variation using the peak areas corresponding to the SIS peptides. Samples to be used for the response curve were prepared in triplicate and each was reconstituted by adding 2 μl of a SIS-peptide solution (from the dilution series described above) and 2 μl of NAT from the working solution (containing 5000 fmol/μl in 3% aqueous ACN/H_2_O) to 46 μl of DBS matrix. Each replicate was injected once for MRM acquisition and three transitions were monitored per peptide. Only the highest intensity and interference-free transition was used for quantification of the LLOQ and for subsequent assay development.

Raw data files were analyzed using Skyline-daily (33), setting the light version of the peptide as the internal standard for normalization. The regression fit was set to linear and the regression weighting was set to 1/x∧2. The MS level was set to 2 and the limit of detection was set to blank plus 3 standard deviations. The automated peak area integration feature in Skyline was used to integrate peak areas. For chromatograms where Skyline consistently failed to select the correct peak boundaries, the boundaries were manually assigned at the points where the chromatogram became parallel with the *x* axis or at the inflection points. The chromatographic peak-area ratio of the heavy to light peptides was plotted against the fmol of SIS peptide (heavy) injected on column. An R-squared value of ≥0.9 was used as the cut-off for an assay to be considered linear. Peak areas were then exported to excel and the coefficient of variation (CV) was calculated at each SIS dilution level. The LLOQ was determined using the peak areas of the SIS peptides with or without normalization, which was dependent on the approach which produced the lower CV. The lowest dilution point displaying a CV of <20% was defined as the LLOQ.

##### Multiday Validation of Assay Analytical Repeatability

For peptides for which an LLOQ was determined, based on the criteria defined above, a multi-day assessment of repeatability was performed, using CPTAC experiment 2 (20) as a guide. Repeatability was assessed using the reverse-curve approach (26, 27), which involves varying the SIS concentration and using NAT and/or endogenous proteolytic peptides as normalizers, or by using the SIS peptide without normalization, for targets for which the NAT peptide was not available or where the endogenous proteolytic peptide was contributing to the variability. DBS matrix spiked with NAT peptides was used for the blank data points which would later be used to assess the limit of detection, defined as three standard deviations higher than the signal measured across the blank samples (three replicates). Repeatability was determined by analyzing 0.2 fmol to 750 fmol of each SIS peptide injected on column. Blank and dilution samples were prepared in three technical replicates - a technical replicate being defined as DBS matrix spiked with NAT and SIS peptides - and each technical replicate was injected once for multiplexed MRM, targeting a single transitions per peptide (the same transition was targeted as was used for determining the LLOQ). These experiments were repeated on five experiment-days with at least 16 h between experiment-days. Targeted MRM and raw data analyses were performed as described in the preceding experimental procedures section, entitled “response curve.” SIS:NAT and/or SIS: endogenous proteolytic peak-area ratios or SIS areas without normalization were used for the calculation of CVs related to the analytical variance. Intra-day CVs were calculated using the three technical replicates at each concentration point on each day, and subsequently averaging across all days. The inter-assay CVs were calculated using the first injection on each day and across all days. This calculation was performed for all three injections, and the resulting CVs were averaged and used as the inter-assay CV. The total variation was calculated as the root sum of squares (RSS) of the intra and inter day assay CVs. This CV was a measure of the analytical variance of the MRM assays.

##### Determination of Assay Selectivity

Assay selectivity was assessed using the CPTAC experiment 3 guidelines (20) with some modification. DBSs obtained from the capillary blood of 6 subjects were processed as described above. A calibration curve for each MRM assay was made by spiking all of the samples with a constant amount of SIS peptide, which served as the normalizer. The lowest point on each calibration curve was the peak-area ratio of endogenous proteolytic peptide/SIS, and three additional higher (concentration) data points were obtained by spiking DBS samples with successively increasing amounts of NAT. The amount of the NAT-peptide spike was a function of the corresponding endogenous proteolytic peptide abundance. The amount of NAT spiked into the sample (equivalent to fmol injected on column) was 1.2 fmol to 3340 fmol for the lowest spikes, 13.3 fmol to 36,740 fmol for the middle spikes and 147 fmol to 404,808 fmol for the highest spikes. The calibration curve in Skyline-Daily was adjusted to assign the sample *without* a NAT spike as the blank, and all other samples were assigned as standards. The slope of the line in each matrix was obtained using only two of the three points in addition to the blank, either the low and medium or the medium and high data points, based on which gave a better (higher) R-squared value. The peak areas selected for determining the slope of the line was kept consistent across the six matrices.

The CPTAC 3 guidelines for analysis of peak-area ratios were modified to include a bias correction in peak areas across the matrices, before calculating the slope. This bias correction was included to compensate for differences in the amount of peptide standard spiked into the samples, which would cause an increase in variance between the calculated slopes of calibration curves when comparing between matrices. To measure the bias between matrices, the NAT/SIS peak-area ratios as measured in each matrix were exported to GraphPad Prism. One matrix was arbitrarily defined as the reference and the other five matrices were compared with the “reference matrix.” The bias across all peptides was calculated using Bland-Altman plots by plotting the NAT/SIS ratio *versus* the average ratio of the two matrices being compared. Peak-area ratios obtained for each concentration point were independently bias-corrected, and the bias-corrected peak-area ratios were then used to calculate the slope in Microsoft Excel using the slope function. The CPTAC experiment 3 guidelines for data analysis require that the slope of the line in an individual matrix be <10% of the average of the slopes across all six matrices. The limitation to this approach is that MRM assays which have a repeatability of >10% could produce a slope of the line, in an individual matrix, that varies more than the cut-off (of 10%) and these assays could falsely be deemed non-selective. To avoid this possibility, assays were deemed to be “selective” if the slopes of the line calculated in each of the six matrices was within two standard deviations of the full process or the analytical assay variance (whichever was higher) of the averaged slope across all six matrices. The difference between the slope in a matrix from the average slope was calculated as a Z-score |(slope_matrix_−slope_ave_)÷σ|, where σ is the full process or the analytical variance. A Z-Score of <2 was indicative of a difference in slope within two standard deviations of the mean across all slopes and was therefore considered to be within the acceptable variance of the assay.

##### Stability Assessment of SIS, NAT, and Endogenous Proteolytic Peptides

Stability testing of the SIS peptides and the corresponding NAT and endogenous proteolytic peptides was performed in accordance with the CPTAC experiment 4 guidelines (20). The DBS matrix (prepared as described above) was spiked with NAT and SIS peptides so that the equivalent of 0.73 fmol (NAT) to 100 fmol (NAT), and 2 fmol (SIS) to 197 fmol (SIS), were injected on column. Aliquots were prepared in 12 sample injection vials, and three of the vials were placed in the autosampler, which was maintained at 5 °C, while the remaining aliquots were stored at −80 °C. Each of the three aliquots on the autosampler was injected in duplicate, giving a total of 6 MRM acquisitions which would later be combined to serve as the 0-h time point. Eight hours after the first injection of the first replicate, the same injection sequence was repeated, and the 6 MRM acquisitions were grouped and used as time point 8–16 h. Thirty five hours after the first injection of the time point zero sample, the injection sequence, MRM acquisitions, and grouping was repeated for time point 35–43 h. After injection of the 8–16 h time point sample, six aliquots from the aliquots stored at −80 °C were thawed at room temperature. Three aliquots were placed back at −80 °C while the remaining three aliquots were used for MRM acquisitions as above. These samples were grouped and designated as a single freeze-thaw cycle. The three aliquots which had been placed back in the −80 °C were thawed a second time and analyzed as above (designated as two freeze-thaw cycles). The three remaining aliquots were removed from −80 °C 4 weeks later and analyzed as above.

The guidelines for data analysis in CPTAC experiment 4 suggest that variability in peak-area ratios should not exceed the variability in peak-area ratios measured at the 0-h time point or the variability assessed in CPTAC experiment 2. The peak-area ratios were obtained setting NAT+ endogenous proteolytic peptides as the normalizer in Skyline-Daily and all peak-area ratios were compared with time point zero using the Z-score which was calculated as follows: |(Peak area ratio_t=0_−Peak area ratio_subsequent t_)÷σ|, where σ is the full process or the analytical variance. A Z-score of <2 was indicative of a difference in peak area ratio within two standard deviations of the peak-area ratio at time point zero and was therefore within the variance of the assay.

##### Assessment of Repeatable Quantification of Endogenous Proteolytic Peptide

Venous blood draws from one male and one female donor were pooled and used to generate DBSs, spotting all five spots on five Whatman 903 protein saver cards. One card was processed per day on five separate days, and each spot was initially processed alone (no pooling) for MRM analysis. The full experiment, from obtaining the venous blood draws through to the MRM analysis of five Whatman 903 protein saver cards over five experiment-days, was performed in two separate experimental blocks (a total of ten experiment-days), in accordance with guidelines outlined in CPTAC experiment 5 (20). Because of repeatedly observed high variance for a subset of proteins (which was attributed to migration in the filter paper), a partial validation of repeatability was performed (in accord with Bioanalytical method validation guidance for industry (38)) by pooling punched DBS spots as follows: the first spot was punched west, the second center, the third south, the fourth north, and the fifth east, and all discs were pooled for processing. A partial validation was performed by processing pooled disc punches from a single Whatman 903 protein saver card, processed in duplicate technical replicates to perform parallel sample processing, over a total of four experiment-days.

Data analysis for calculation of intra and inter-assay CVs was as follows; peak-area ratios were measured using Skyline-Daily, setting the SIS as the normalizer and endogenous proteolytic peptide as the numerator. Peak-area ratios were obtained from technical replicates which were each used for triplicate MRM analysis (six total injections per experimental day). Peak-area ratios from triplicate injections were averaged and the intra-day CV was calculated using the average peak area ratio obtained for technical replicates on each day. The CVs across all 4 days were averaged to obtain the average intra-assay CV. The inter-day CV was calculated as follows; the peak area ratios from three replicate injections for each technical replicate were averaged. The average peak rea ratio of technical replicate one, on each day, was used to calculate an inter-day CV, and the same calculation was performed for technical replicate two (across the four experimental days). The resulting inter-day CVs were averaged to obtain an average inter-day CV. The total variation was calculated as the RSS of the average intra and inter-day CVs.

##### Protein Stability Testing in DBS

Protein stability in DBSs was tested at three temperatures and up to 57 days of storage prior to processing. A pooled venous draw from 5 donors was used to spot all five spots of Whatman 903 protein saver cards using 50 μl of blood per spot and spotting a total of 46 cards (230 spots). After drying overnight, each card was individually placed in a heat sealable bag with desiccant and sealed. Bags were placed in cardboard freezer boxes and stored at either −20 °C, ambient laboratory temperature (∼25 °C), or 40 °C. After 24 h of storage, one card from each temperature was processed and analyzed using MRM. These samples served as time point zero and all subsequent peak-area ratios measured at future time points would be compared with this time point to assess whether a protein concentration had a measurable change after storage. The subsequent time points measured were Days 3, 4, 7, 13, 21, 28, 35, 42, and 57. Peak-area ratios (endogenous proteolytic peptide/SIS), measured at each time point, were compared with time point zero using the Z-score which was calculated as follows: |(Peak area ratio_Day1_−Peak area ratio_Day n_)÷σ|, where σ is the full process or the analytical variance. A Z-score < 2 was indicative of a difference in peak area ratio within two standard deviations of the peak area ratio at Day 1 and therefore within the variance of the assay.

##### Determination of Correction Factors

A correction factor for each DBS MRM assay was determined to convert the fmol of peptide measured on column from a DBS sample, to a concentration in whole blood. To determine these correction factors, MRM assays were performed to measure endogenous proteolytic peptide/SIS peak-area ratios in whole-spot DBS, 6 mm disc punched DBS, whole blood, and lyophilized blood (in parallel). Pooled blood was used to generate DBSs by spotting either 50 μl or 13 μl of blood on Whatman 903 protein saver cards, each in triplicate. Whole blood in the liquid phase was processed, adding 13 μl directly to 237 μl of extraction buffer (2% DOC in 50 mm ammonium bicarbonate), in triplicate. In parallel, 13 μl of blood was lyophilized in triplicate. The 50-μl DBSs were used to punch out 6-mm discs using a pneumatic punch, whereas the 13-μl DBS spots were cut out whole. Each spot was processed without pooling by performing solid to liquid phase extraction in 250 μl of extraction buffer. Similarly, the lyophilized blood samples were each processed using 250 μl of extraction buffer. Samples were then processed for MRM analysis as described in the previous sections. Data were analyzed in Skyline-Daily as described in the previous sections, and the endogenous proteolytic peptide/SIS peak-area ratios were used to calculate the correction factors. The measured peak-area ratios in each liquid blood technical replicate was divided by the corresponding peak-area ratio in each 13-μl whole-spot DBS technical replicate and averaged across all three ratios to give a unique correction factor for each MRM assay. Using the identical approach, MRM assay-specific correction factors were calculated for the whole spot and for 6-mm disc punched DBS samples. Finally, to make the 6-mm disc punch into a volumetric sample processing strategy, the whole spot-to-disc punch correction factor was divided by the whole spot-to-blood correction factor. Using the resulting correction factors provides the option of reporting protein concentration per volume of blood even though the concentration measurement was performed using a 6-mm disc punch from a DBS sample. The advantage of this is that volumetric spotting on Whatman 903 filter papers would not then be needed - instead, blood could be sampled directly from a capillary puncture. The identical data analysis was performed using lyophilized blood to determine whether the drying process was the main contributing factor to the higher concentration measurements that was observed in DBS relative to whole liquid blood samples.

##### Calibration Curves for Measuring Peptide Concentration

The concentrations of the endogenous proteolytic peptides were measured using the linear regression equation for the line obtained by generating calibration curves in a surrogate matrix background. The surrogate matrix has been described in the experimental procedures section entitled pre-analytical sample preparation. The regression equation was generated by plotting NAT/SIS ratio on the *y* axis and the NAT concentration on the *x* axis. Wherever possible, the SIS peptide concentration was adjusted to fall within a factor of 10 of the concentration of its endogenous proteolytic peptide counterpart. The NAT peptide was used to make a dilution series to give a seven-point calibration curve with a three-orders-of-magnitude dynamic range, covering the endogenous proteolytic peptide concentration range. Calibration curves were generated using Skyline-Daily by setting the regression fit and weighting to linear and 1/x*x, respectively. Using the equation of the line and the endogenous proteolytic peptide/SIS peak-area ratio, the fmol of endogenous proteolytic peptide injected on column can be calculated for an unknown sample. Finally, the concentration of the peptide in the original blood sample can be calculated using the correction factor for each analyte, as described in the experimental procedures section entitled Determination of Correction Factors.

Details of the donor cohort and collection of capillary blood from finger sticks are described in the Experimental Procedures sections on collection of human whole blood and pre-analytical sample preparation. DBS samples were processed within 13 days after collection (of capillary blood as DBS) as described in the experimental procedures section on pre-analytical sample preparation. MRM analysis was performed on each sample and the measured protein concentration was the average of three MRM analyses.

## RESULTS

### 

#### 

##### Protein Identification and Selection for MRM Assay Development

As the first phase in a program to develop a comprehensive assay for quantifying proteins from DBS, proteins were selected for MRM assay development based on their detectability in DBSs and by consulting the literature on other studies which used derivatives of blood, such as purified red blood cells or plasma. Proteins in DBSs were identified without depletion of high-concentration proteins, using a routine bottom-up proteomic workflow via data dependent acquisition (DDA) which identified 295 unique proteins (supplemental Table S1). The list of protein targets was extended to 2650 proteins based on earlier studies which utilized depletion of high-concentration proteins and up-stream fractionation prior to bottom-up proteomics (39, 40). Proteolytic peptides to target a comprehensive panel of plasma and cellular proteins were then selected using PeptideTracker, (29) which narrowed the list of protein targets to 270 proteins, represented by 334 SIS peptides (supplemental Table S1). Thus, the number of proteins targeted for MRM assay development was limited by our throughput for synthesizing and characterizing synthetic peptide standards.

##### Measurement of Lower Limit of Quantification and Linear Range

The LLOQ and the range of linearity for the SIS peptides were determined in a DBS matrix based on seven-point response curves using the reverse-curve strategy (26, 41), *i.e.* the concentration of stable isotope labeled standard (SIS) peptides is varied and the corresponding unlabeled standard (NAT) and/or endogenous proteolytic peptides are used as normalizers. Linearity was studied over a dynamic range of four orders of magnitude, and a response curve was linear if the R-squared value was greater than 0.90 (supplemental Fig. S3*A* and supplemental Table S1). A 20% CV cutoff was used to define the LLOQs, which are presented in supplemental Fig. S3*B* as a histogram showing the number of peptide LLOQs determined at each concentration tested. Using these criteria, LLOQs were determined for 315 of the original 334 SIS peptides (representing 257 proteins), with concentrations reaching as low as 0.1 fmol, with a median of 4 fmol, and 75% percentile of ≤11 fmol injected on column (supplemental Table S1). The 21 peptides for which an LLOQ could not be established were not considered for further assay development.

##### Repeatability of MRM Assays

**Fig. 1.**
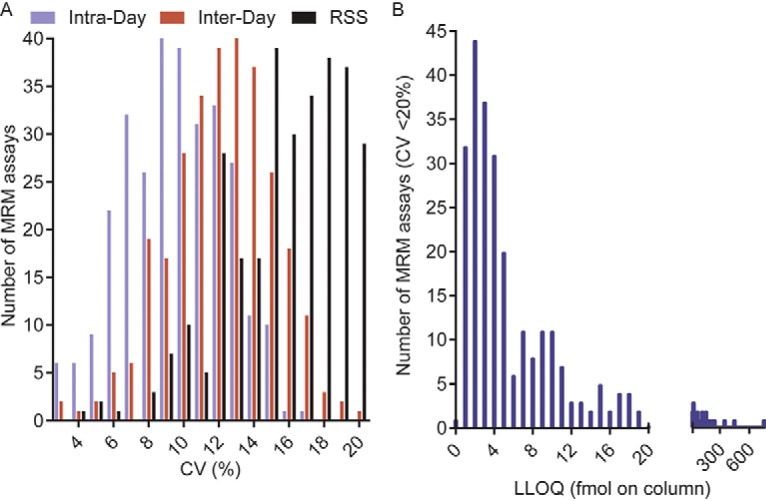
**LLOQ precision across five experiment-days.** *A*, The MRM assay precisions at the LLOQ are depicted as a frequency histogram showing the number of MRM assays and corresponding intra-day, inter-day, and total CVs, which are shown as blue, red and black bars, respectively. *B*, The frequency of MRM assays at each concentration representing the LLOQs, are depicted as a frequency histogram.

MRM assay repeatability at the LLOQ was determined for the 315 MRM assays for which an initial LLOQ was measurable. Assay repeatability was determined by analyzing three technical replicates per day, on five distinct experiment-days, allowing the determination of intra- and inter-day CVs. The total CV was calculated as the root sum of squares (RSS) (of the intra- and inter-day CVs). Assay repeatability was tested at the LLOQ and at higher concentrations (up to 3 orders of magnitude), and for 298 MRM assays, corresponding to 244 unique proteins, a concentration at which the total CV was below 20% could be determined (Fig. 1*A* and 1*B* and supplemental Table S1). The repeatability of the assays is shown in Fig. 1*A* as a frequency distribution of the measured CVs. Across the 298 MRM assays, the median intra-day, inter-day, and total CVs at the LLOQ were 10%, 12%, and 18%, respectively, with a range of 3–20%. The measured LLOQs are presented in Fig. 1B as a frequency distribution of the number of MRM assays and the corresponding LLOQs. The median LLOQ across the 298 MRM assays was 5 fmol of peptide injected on column, and 75% of the MRM assays had LLOQs of ≤13 fmol injected on column, whereas the lowest and highest measured LLOQs were 0.2 fmol and 750 fmol of peptide injected on column. The remaining 25 peptides, which demonstrated a total CV of >20% at all tested concentrations, were not considered for further assay development.

##### MRM Assay Parallelism

**Fig. 2.**
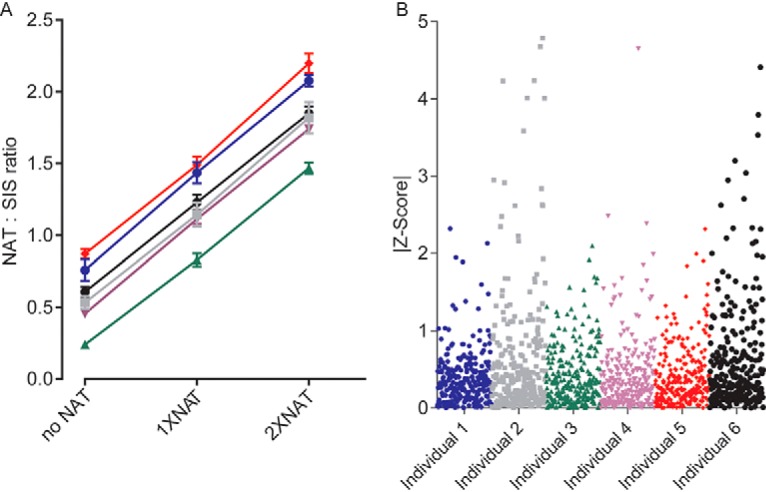
**Assessment of assay parallelism in six biological replicates of DBS matrix.** *A*, The resulting calibration curves, targeting the peptide VSVFVPPR, are displayed as an example. The normalized peak-area ratios were plotted on the *y* axis and the concentrations were plotted on the *x* axis by assigning the endogenous proteolytic peptide in each matrix as zero and the NAT peptide concentrations based on the amount spiked. *B*, The calculated absolute values of Z-Scores for 258 MRM assays (218 unique proteins) are plotted on the *y* axis and each unique matrix (individual) is plotted on the *x* axis. The difference between the slope in a matrix from the average slope was calculated as a Z-score |(slope_matrix_−slope_ave_)÷σ|, where σ is the full process or the analytical variance. A Z-Score of <2 was indicative of a difference in slope within two standard deviations of the mean across all slopes, and was therefore considered to be within the acceptable variance of the assay.

Interference and matrix-effect testing (“assay parallelism”) was performed using DBS matrix obtained from six individuals. Parallelism was assessed by generating calibration curves in each of the six DBS matrices, using SIS as the normalizer and varying the concentration of NAT. The lowest point in each calibration curve was the endogenous proteolytic peptide/SIS peak-area ratio for a sample not spiked with NAT. The slope of the calibration curve across the six matrices was compared to assess parallelism (Fig. 2*A*). An assay was considered as showing parallelism if the measured slope (in an individual matrix) varied from the average slope across all six individuals by less than two standard deviations. Parallelism was determined for 258 MRM assays (*i.e.* assays for which NAT was available), corresponding to 218 proteins, of which 225 MRM assays displayed parallelism as defined above (Fig. 2*B* and supplemental Table S1).

The 30 assays which were *not* found to be interference-free were still considered for further assay development and the cause of interference and how it will be overcome will be the subject of a future study.

##### Peptide Standard and Endogenous Proteolytic Peptide Stability Testing

**Fig. 3.**
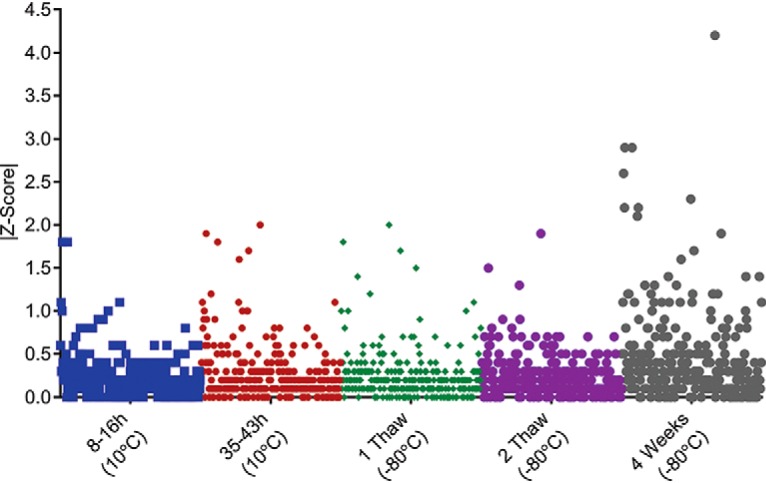
**Peptide standard and endogenous proteolytic peptide target stability when stored in-solution.** DBS matrix was spiked with SIS and NAT peptide standards and MRM performed at time zero, at 8–16 h on the autosampler, at 35–43 h on the autosampler, after one freeze/thaw cycle, after two freeze/thaw cycles and after storage at −80 °C for 4 weeks. SIS-normalized peak-area ratios were compared with time zero using MRM assay specific Z-Scores. Peak-area ratios were compared with time point zero using the Z-score which was calculated as follows: |(Peak area ratio_t=0_−Peak area ratio_subsequent t_)÷σ|, where σ is the full process or the analytical variance. A Z-score of <2 was indicative of a difference in peak area ratio within two standard deviations of the peak-area ratio at time point zero, and was therefore within the variance of the assay.

DBS samples prepared for MRM analysis (*i.e.* spiked with SIS and NAT), were used to determine whether prolonged storage on the auto-sampler or storage in the freezer could affect assay repeatability. Assay repeatability was tested by comparing the variance in the SIS:NAT, SIS: endogenous proteolytic peptide, or SIS:NAT+ endogenous proteolytic peptide peak-area ratios at each time point to the variance at time zero (Fig. 3). A change in peak area ratio of greater than two standard deviations (Z-Score > 2), from time zero, was taken to indicate that the standard and/or endogenous proteolytic peptide(s) were not stable on the autosampler and/or after exposure to freeze/thaw cycles. Across all time points and freeze/thaw cycles, for the 294 MRM assays (targeting 240 proteins) tested, 283 had variabilities below the cut-off (Z-Score of ≤2) (Fig. 3 and supplemental Table S1). Storage of DBS samples prepared for MRM analysis (*i.e.* spiked with SIS and NAT), for 4 weeks affected repeatability the most, with 8 MRM assays displaying Z-scores of >2 (Fig. 3 and supplemental Table S1).

##### Repeatability of Endogenous Proteolytic Peptide Quantification

**Fig. 4.**
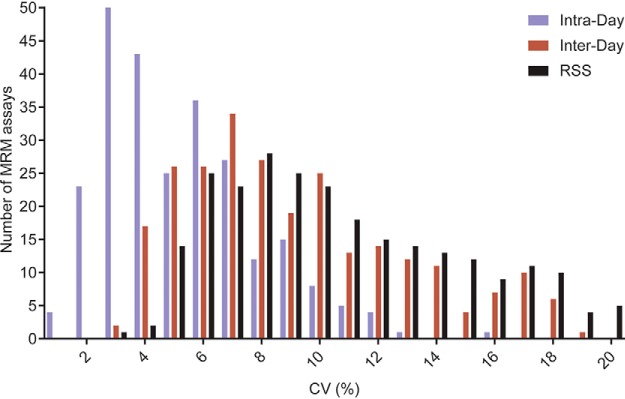
**Multi-day assessment of repeatability for quantification of endogenous proteolytic peptide.** DBS samples were processed and analyzed via MRM on four experiment-days and the intra-day, inter-day and total CVs determined using peak area ratios of endogenous proteolytic peptide:SIS. The data is presented as a frequency histogram of the number of MRM assays and the corresponding CV, plotted on the *x* axis.

DBSs were generated using pooled venous blood, donated by one male and one female subject, and were subsequently processed for MRM assays targeting 243 proteins. Measurement of endogenous proteolytic peptide:SIS peak-area ratios showed that all 243 proteins could be detected and 213 proteins could be reproducibly quantified (Fig. 4). Multi-day assessment of the quantification of the endogenous proteolytic peptides, used for quantification of the endogenous proteins, showed that 213 proteins could be quantified with an intra-day CV of <20%, 198 proteins with an inter-day CV of <20%, and 193 proteins with a total CV of <20%, calculated as the root sum of squares of the intra-day and inter-day CVs (Fig. 4 and supplemental Table S1).

##### Assessment of Protein Stability in DBSs

**Fig. 5.**
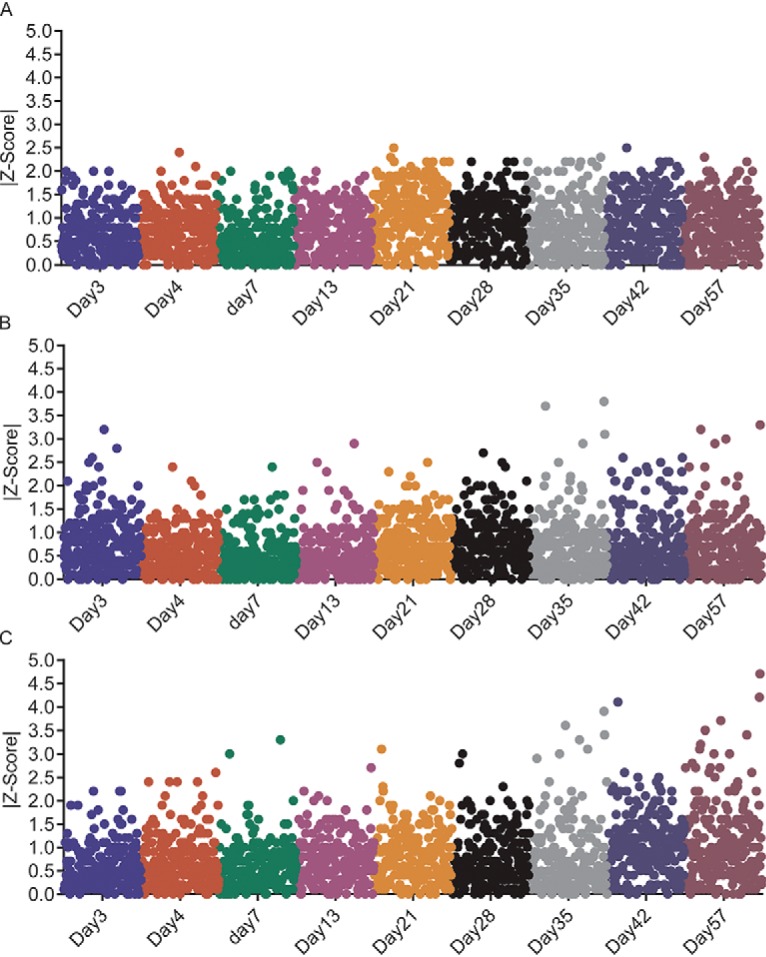
**Multi-week assessment of protein stability in DBS.** The stability of proteins in DBS was determined at ambient laboratory temperature, at −20 °C, and at +40 °C. Panels A-C display the results for the three different storage temperatures, ambient laboratory temperature, −20 °C, and +40 °C, in that order. The absolute values of the Z-Scores are plotted on the *y* axis for each day, which are plotted on the *x* axis.

The stability of proteins in DBSs stored at ambient laboratory temperature, at −20 °C, and at +40 °C, was tested for up to 57 days to determine whether cold-chain logistics could be eliminated for shipping DBS samples (Fig. 5). A comparison of the endogenous proteolytic peptide/SIS peak-area ratios to Day 1, using MRM assay specific Z-Scores, indicated that 95% of the proteins were stable for 57 days when stored at ambient laboratory temperature and at −20 °C, and up to 28 days when stored at +40 °C (Fig. 5). As shown in Fig. 5, a Z-Score below 2 indicates that the endogenous proteolytic peptide/SIS peak-area ratio at the designated time point was < 2 standard deviations different from the endogenous proteolytic peptide/SIS peak area ratio measured at Day 1. In other words, any change in peak-area ratio (when compared with Day1) could be attributed to the variability of the assay itself, and not to the stability of the protein. Conversely, a Z-Score of >2 was an indication that protein stability had been compromised during storage.

##### Correction Factors for Measuring Protein Concentrations in DBSs

To report concentration of protein per volume of blood (as opposed to per DBS punch), correction factors were calculated to convert the concentration values in DBSs to those in whole blood (supplemental Table S1). Comparison of endogenous proteolytic peptide/SIS peak-area ratios, for 225 peptides, in matched blood and DBSs showed higher overall mean values in the latter, with a mean of 1.26 and a minimum and maximum range of 0.5 and 6.2 fold, respectively (supplemental Fig. S4). To determine whether the drying process increased the proteolytic digestion efficiency, matched blood and lyophilized samples were used to compare the endogenous proteolytic peptide/SIS peak-area ratios between the samples (supplemental Fig. S4), resulting in higher endogenous proteolytic peptide/SIS peak area ratios in lyophilized samples, with a mean of 1.20 and a minimum and maximum range of 0.5 and 3.8 fold, respectively. A comparison of DBSs with lyophilized blood (supplemental Fig. S4) showed endogenous proteolytic peptide/SIS peak area ratios closer to 1, with a mean of 1.06 and a minimum and maximum range of 0.5 and 2, respectively, which suggested that drying prior to sample processing was the main factor in increasing the tryptic digestion efficiency.

Higher ratios of endogenous proteolytic peptides/SIS in DBS and lyophilized blood were not always observed; for some endogenous proteolytic peptides, the ratios to SIS counterparts were higher in blood. These results suggested that the drying process may have negatively affected subsequent protein solubility and/or recovery from DBSs during the solid-to-liquid-phase extraction.

##### Protein Concentration Measurements in DBS Sampled Capillary Blood

Protein concentration measurements were performed using calibration curves which were generated in a surrogate matrix spiked with SIS and NAT peptide standards as the normalizer and variable, respectively. Calibration curves were generated for the quantification of 252 peptides (determined by the availability of the synthetic NAT peptide), corresponding to 215 unique proteins. The quality of the calibration curves was assessed using R-squared values, accuracy (for calculating the concentration of NAT), and the number of data points used to generate each calibration curve, all of which are presented as frequency histograms in supplemental Fig. S5*A*–S5*C*. As illustrated in supplemental Fig. S5*A*, 252 calibration curves displayed good linearity, across a NAT peptide concentration range of up to 3 orders of magnitude, with median R-Squared values of 0.9959, and maximum and minimum R-Squared values of 1 and 0.9541, respectively. Calibration curves accurately measured the concentrations of samples used for generating data points (because known concentrations of NAT were added) as shown in supplemental Fig. S5*B*, with median accuracies of 100.4%, and maximum and minimum accuracies of 139.1% and 66.2%, respectively. The number of data points used for generating each calibration curve is also shown in supplemental Fig. S5*C*, which indicates a median of 7 data points per calibration curve, a maximum of 14 data points, and a minimum of 3 data points per calibration curve.

**Fig. 6.**
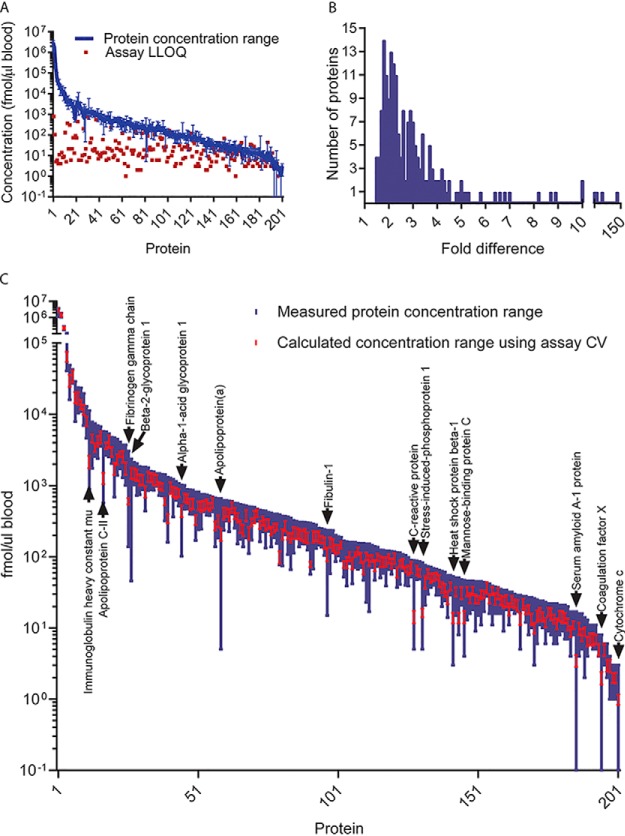
**Inter-individual protein concentration ranges in DBS.** *A*, The concentrations for 201 proteins measured in all 20 individuals are plotted on the *y* axis in fmol/μl. MRM assay LLOQs are also plotted (in red). *B*, The biological variability or inter-individual protein concentration variability is also presented as frequency histogram of the concentration across all twenty individuals. *C*, The biological/inter-individual variability (blue) is shown as a measured protein concentration across the 20 individuals. The median concentration ± standard deviation, specific for each assay, is superimposed in red.

Using calibration curves and correction factors, the concentration ranges of protein targets were established using capillary blood DBSs taken from a cohort of 20 healthy (self-reported) same-race males, ages 19–39 (Fig. 6*A*). Measurement of protein concentrations in capillary blood of these individuals revealed intra-individual concentration ranges between proteins spanning 6 orders of magnitude, as well as differences in protein concentrations between individuals (Fig. 6*B*). The assay-specific LLOQs are plotted on the same graph for comparison (Fig. 6*A*) and show that the majority of protein targets were quantified above their LLOQs. Eight protein targets were found at levels slightly below their determined LLOQs (LLOQ: endogenous proteolytic peptide, fmol/μl blood) (119:90, 68:42, 42:34, 162:155, 27:21, 34:29, 32:27, 32:29).

The discriminatory power of the developed MRM assays was visualized by superimposing the measured protein concentration range (across the 20 individuals) over the theoretical maximum and minimum variance from the median, which were calculated using MRM (protein) assay-specific variances (Fig. 6*C*). It can be observed, from Fig. 6*C*, that the theoretical protein concentration ranges were within the measured ranges, *i.e.* the MRM assay variance was low enough to discriminate protein concentration differences between healthy, same-race, same-sex, and similar-age individuals. Inter-individual protein concentration variability is shown in Fig. 6*B* and 6C, with a median concentration range difference for a specific protein of 2.6 fold, a maximum of 132.2 fold (Apolipoprotein(a)), and a minimums of 1.5 fold (Fig. 6*C*). The 14 proteins that displayed a fold-difference of greater than 10 in concentration range are labeled in Fig. 6*C*.

## DISCUSSION

The goal of the present study was to develop assays for precise and multiplexed protein concentration measurements compatible with DBS sampling and to determine concentration ranges in a sample of age matched, same sex, and same race individuals. The median lower limit of quantification across 315 peptides was 5 fmol on column, approximately equivalent to 16.7 fmol of protein per μl of blood. When converted to mass per ml of blood, the median LLOQ was equivalent to 0.083 μg/ml, 0.996 μg/ml or 8.3 μg/ml for proteins with molecular weights of 5 kDa, 60 kDa, and 500 kDa, respectively. The lowest LLOQ measured was 0.2 fmol on column which demonstrated a limit of quantification equivalent to ∼0.003–0.332 μg/ml of protein in blood. For comparison, the typical protein concentrations in plasma for interleukins, tissue leakage proteins, and classical plasma proteins are < 0.0001 μg/ml, <1 μg/ml, and <40 000 μg/ml, respectively (42, 43). Thus, the quantitative sensitivity of MRM on DBS samples would allow the measurement of protein concentrations of tissue leakage and classical plasma proteins, but not interleukins.

In addition to offering relatively high sensitivity, the calibration curves generated from the MRM assays displayed linearity over a concentration range covering 3 orders of magnitude. Considering the largest inter-individual concentration range observed in this study (∼130 fold for Apolipoprotein(a)), the inter-individual or biological variability was quantifiable within the linear range of the assays. Yet another apparent advantage of the MRM assays was the quantitative precision which appeared high enough to discriminate protein concentrations in a same race, same sex, and similar age population. Based on these results, it should be possible to use MRM assays for measuring protein concentrations in DBSs to establish reference ranges in healthy and or “control” populations, and for establishing unique Z-Scores that could discriminate healthy and diseased individuals.

MRM assays were further validated by measuring the analytical responses in DBS matrices obtained from different individuals, and by assessing the stability of peptide standards during sample analysis and storage in a liquid format. Not all the MRM assays could be tested for parallelism, because of the lack of available NAT at the time these experiments were performed. Of the 258 assays tested for parallelism, the majority displayed parallelism, thus confirming that the assays were interference free and showed minimal matrix effects, which indicated that the concentration measurements of the corresponding proteins would be relatively accurate and specific, regardless of the individual being measured.

The peptide standards were found to be stable throughout the full assay workflow and for up to 2 days on the autosampler at 5 °C, and after multiple freeze/thaw cycles. A slight increase in variability was observed after 4 weeks storage at −80 °C which was attributed in part to performance of the LC system, as there was a retention time shift across all peptides being measured at that specific time point.

As alluded to earlier, one of the main advantages of DBS sampling is the increase in analyte stability which can result in improved accuracy of the concentration of the analyte measured in blood, and could also reduce shipping and storage costs by eliminating temperature-controlled shipping and storage. This would allow sampling in remote locations and analysis of the DBS sample later.

Although proteins are generally more stable in dried format, compared with in solution, the drying process induces conformational changes in protein structure (14) that may affect downstream trypsin digestion efficiency. Differences in digestion efficiencies could lead to different measured protein concentrations from the same sample, when measured in liquid blood *versus* DBSs. Indeed, comparison of endogenous proteolytic peptide concentrations measured in matched blood and DBS samples consistently resulted in an overall higher mean concentration when using DBSs. Interestingly, similar comparisons (in the present study) between matched blood and lyophilized blood resulted in overall higher mean concentration measurements in the latter. It was concluded that the drying process was the main contributing variable that led to differences in protein concentrations. However, not all of the endogenous proteolytic peptides followed this trend and for some of the endogenous proteolytic peptides, the concentrations were measurably higher in blood (compared with matched DBS and lyophilized samples), suggesting that protein recovery and/or solubility after the drying process also adds variability to concentration measurements. Based on these variabilities, correction factors are needed in order to express concentrations per volume of blood when measurements are made in DBS. The correction factors determined were unique for each protein target and, in addition to controlling for digestion efficiencies, also controlled for variations in recoveries and solubility.

Using the derived correction factors, endogenous protein concentrations were measured using MRM assay-specific calibration curves which were generated using a SIS normalizer while varying the NAT concentration. Calibration curves were generated using external calibration rather than the internal calibration strategy because the former only slightly underperforms compared with the latter (average difference in slope was 3.8%), but is more cost effective when a large number of samples are analyzed (44).

Protein Stability Testing at Three Different Temperatures—at ambient laboratory temperature, at −20 °C, and at +40 °C—confirmed the hypothesis that the target proteins remained stable in the DBS format even at extreme temperatures. Storage at 40 °C for 57 days, however, resulted in significant differences compared with day 1, in that 26 proteins displayed higher concentrations at day 57, indicating that storage or exposure of DBS samples to elevated temperatures over the course of several weeks will result in increased concentration measurements, possibly because of more efficient extraction and/or higher digestion efficiency. Multi-week exposure of DBS samples to elevated temperatures will therefore decrease quantitative precision. Based on these results, we conclude the cold chain logistics can be eliminated for the shipping and storage of DBS samples. For storage up to 2 months, ambient laboratory temperature appeared to provide the best quantitative repeatability.

In summary, 200 MRM assays have been developed for precise protein quantification of endogenous proteins in DBS. These validated assays may find application in *de novo* identification of protein biomarkers or for population-wide screening of early protein markers of disease.

## DATA AVAILABILITY

Skyline files pertaining to assay development are available from the Panorama Public portal, (https://panoramaweb.org/yfoOuC.url). The ProteomeXchange ID for this data is PX ID: PXD015118 (http://proteomecentral.proteomexchange.org/cgi/GetDataset?ID=PXD015118). Untargeted proteomics data have been deposited in MassIVE (MSV000084629) (https://massive.ucsd.edu/ProteoSAFe/dataset.jsp?task=9ac8769ffb2e4d7b90bbfd0d679d68a4).

## Supplementary Material

Suplemental Table S1

Supplemental figures

## Footnotes

^1^ The abbreviations used are:

DBS: dried blood spot
MRM: Multiple reaction monitoring
CPTAC: Clinical Proteomics Tumor Analysis Consortium
LLOQ: lower limit of quantification
DOC: sodium deoxycholate
SIS: stable isotope labeled standard
RSS: root sum of squares.
