# Correction: Concentration Determination of >200 Proteins in Dried Blood Spots for Biomarker Discovery and Validation

**DOI:** 10.1074/mcp.AAC120.002331

**Published:** 2020-11-25

**Authors:** Azad Eshghi, Adam J. Pistawka, Jun Liu, Michael Chen, Nicholas J.T. Sinclair, Darryl B. Hardie, Monica Elliott, Lei Chen, Rachael Newman, Yassene Mohammed, Christoph H. Borchers

VOLUME 19 (2020) PAGES 540-553

There was an error in the grant information in which a grant acknowledgment was omitted. The Partnership for Clean Competition (Colorado Springs, CO, USA) should be added to this section.

