# Supplementary material for: Concentration Determination of >200 Proteins in Dried Blood Spots for Biomarker Discovery and Validation
Source: Mol Cell Proteomics. 2020 Jan 1;19(3):540–53. doi: 10.1074/mcp.TIR119.001820 (PMC7050112; doi:10.1074/mcp.TIR119.001820)
Supplement: Supplemental figures [file 156416_1_supp_433578_q23vym.docx]

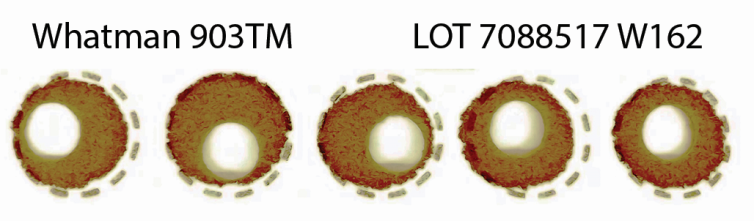


**supplemental Fig.S1. Sampling from DBS via 6 mm disk punch.** Capillary blood was obtained from finger punctures into vacutainer tubes and used to volumetrically spot 50 µl of blood in each of 5 Whatman 903 protein saver card spots. DBS was sampled by punching 6 mm diameter using a pneumatic punch. DBS samples were punched West, South, East, North and Centre and processed for MRM analysis.


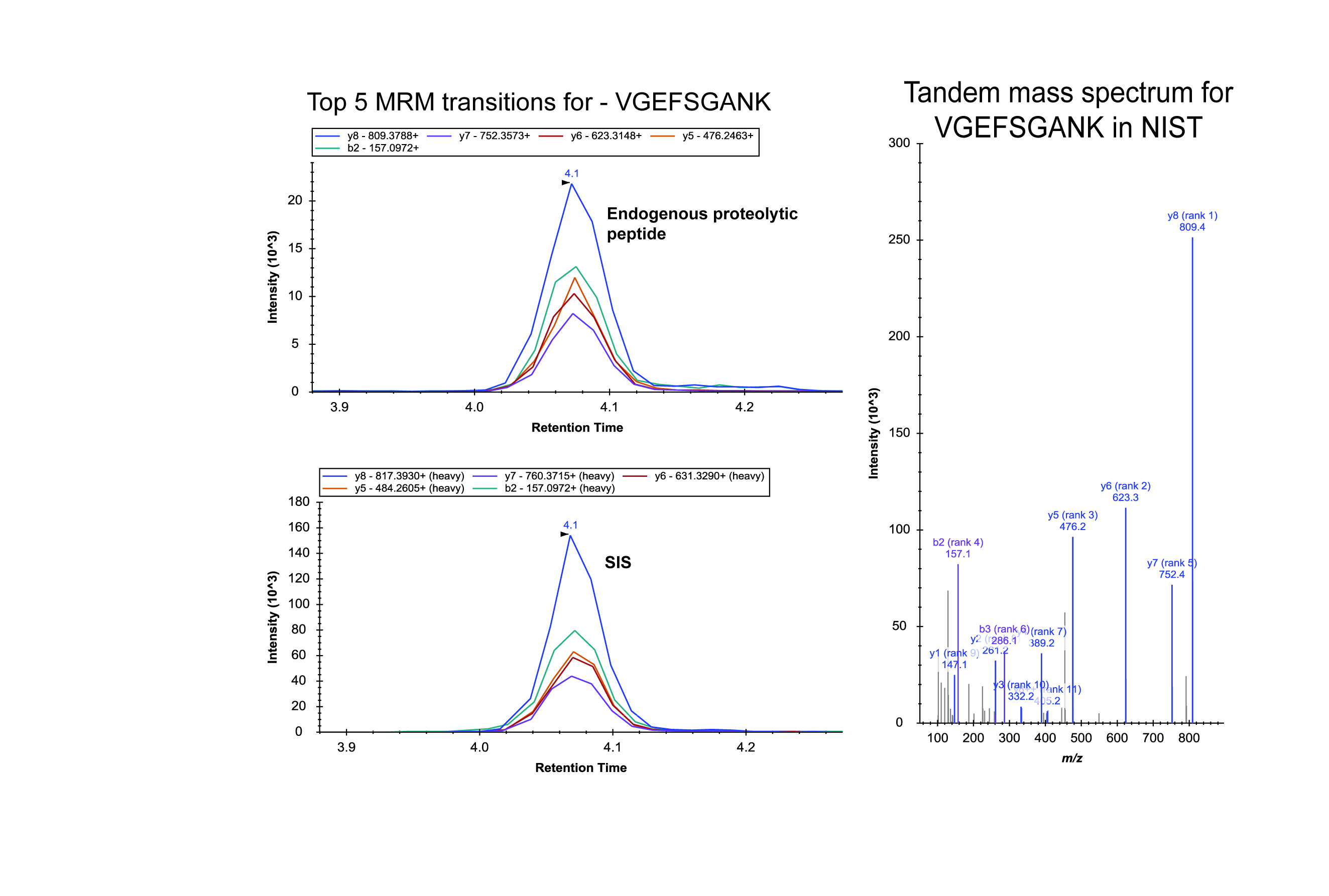


**supplemental Fig.S2. Comparison of MRM and tandem mass spectra for initial selectivity assessment.** As an example, the top five transitions representing the proteotypic peptide (VGEFSGANK), for redox signaling protein thioredoxin (P10599) are presented. Comparison of product ion transitions between SIS and endogenous proteolytic peptide indicates nearly identical fragmentation behavior, which is a confirmation of selectivity. All product ions corresponding to the parent ion are also present in the NIST tandem mass spectrum, which is further confirmation of selectivity.


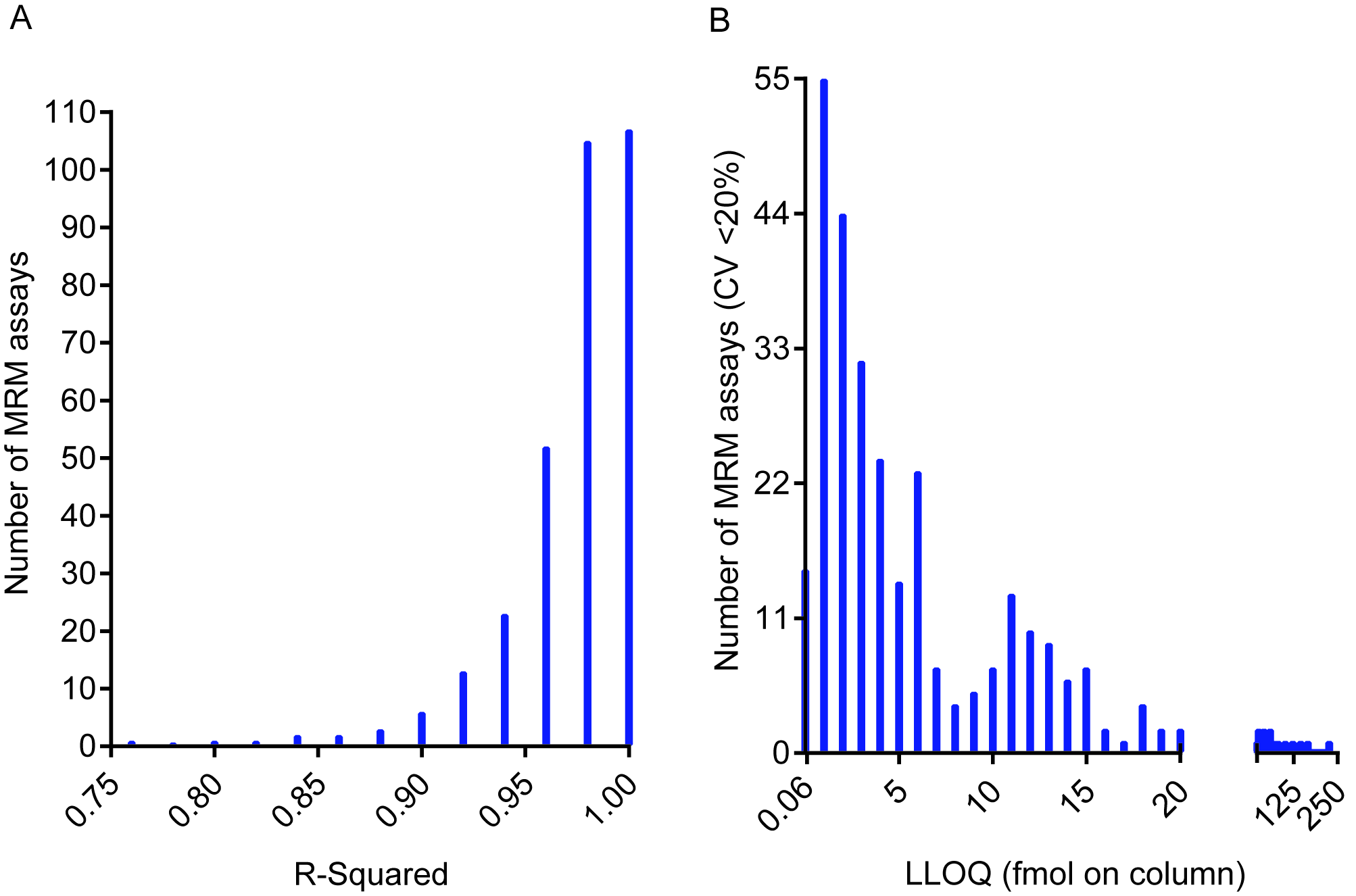


**supplemental Fig.S3. MRM assay linearity and** **lower limits of quantification (LLOQs) in DBS matrix**. MRM assay linearity and lower limits of quantification were tested in DBS matrix using reverse curves, i.e., varying the concentrations of the SIS standards, with or without normalization using corresponding NAT and/or endogenous proteolytic peptides. **A**. The linearity of the MRM assays are presented as a frequency histogram displaying the R-squared values and the frequency of MRM assays. Linearity was tested spanning a concentration of four orders of magnitude and the R-squared values reflect the linearity over the whole range. An assay was considered to be linear if R-squared was >0.95. For assays with R-squared less than 0.95, the dynamic range of the calibration curve was reduced. **B**. MRM assay specific LLOQs are presented as frequency histogram displaying the frequency of MRM assays at each concentration. The LLOQ was determined using a CV cut-off of ≤20% (N=3). The lowest achievable LLOQ was 0.1 fmol, with a median of 4 fmol and 75% percentile of ≤11 fmol injected on column.


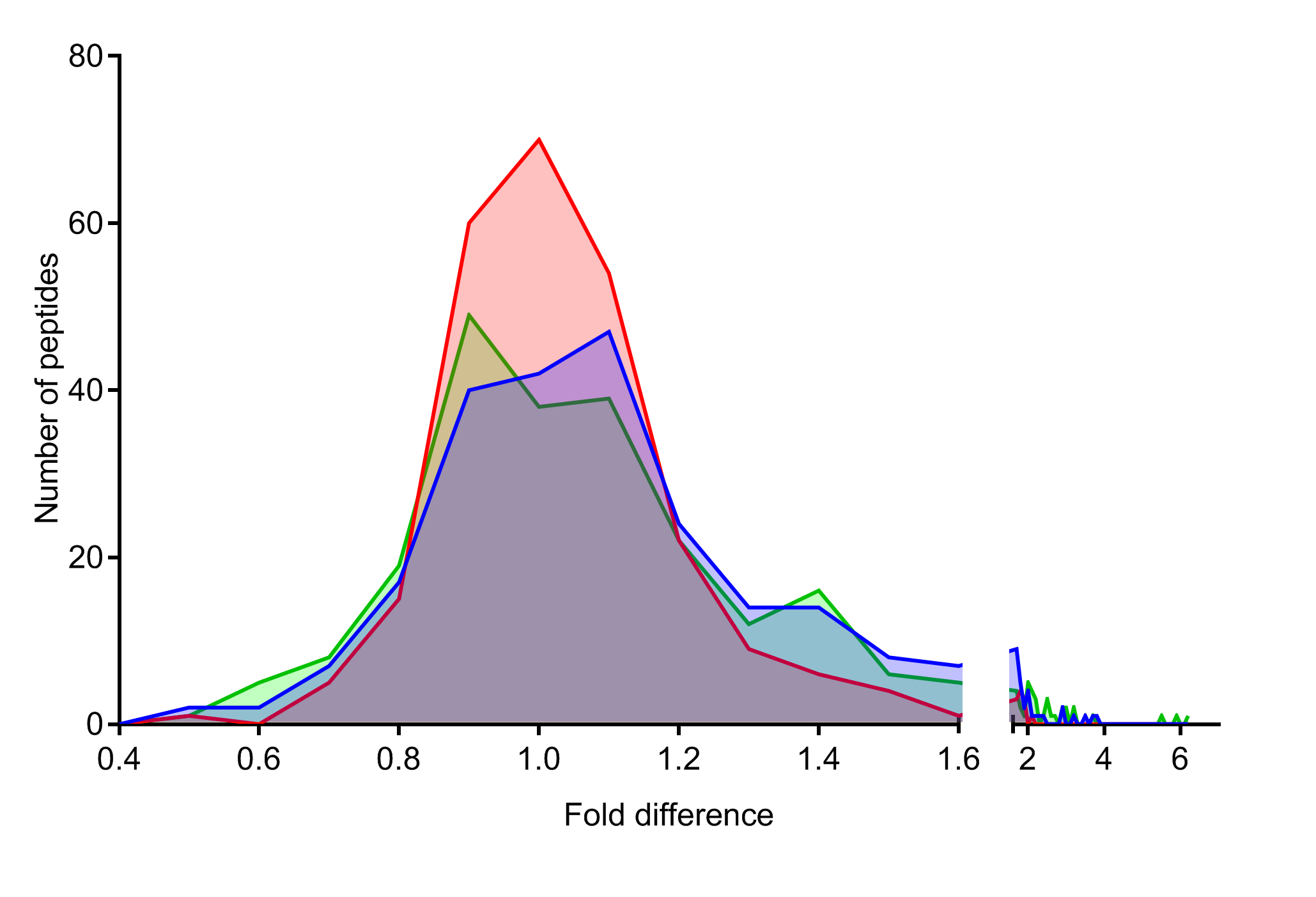


**supplemental Fig.S4.** **Effects of DBS sampling on quantitative performance of MRM assays**. The quantitative performance of MRM assays was compared using liquid blood, DBSs, and lyophilized blood. Triplicate samples of liquid blood, DBSs, and lyophilized were processed and analyzed via MRM comparing peak-area ratios of endogenous proteolytic peptide : SIS. The data for 255 peptides is presented as a continuous frequency histogram with the number of peptides plotted on the y-axis and the fold difference in peak-area ratios plotted on the x-axis. The green graph compares DBS to blood, the blue graph compares lyophilized blood to blood, and the red graph compares DBSs to lyophilized blood. The latter graph shows a pronounced peak centering around unity with data nearly equally distributed to the left and to the right of unity. This was reflective of similar endogenous proteolytic peptide : SIS peak-area ratios measured in DBSs and lyophilized blood which displayed lower and upper 95% confidence intervals of 1.03 and 1.09-fold higher in DBSs than in lyophilized blood samples. In contrast, both the green and blue graph are skewed to the right, and relatively flat at the peak, which indicate higher peak-area ratios measured in DBSs and lyophilized blood, when compared to blood.


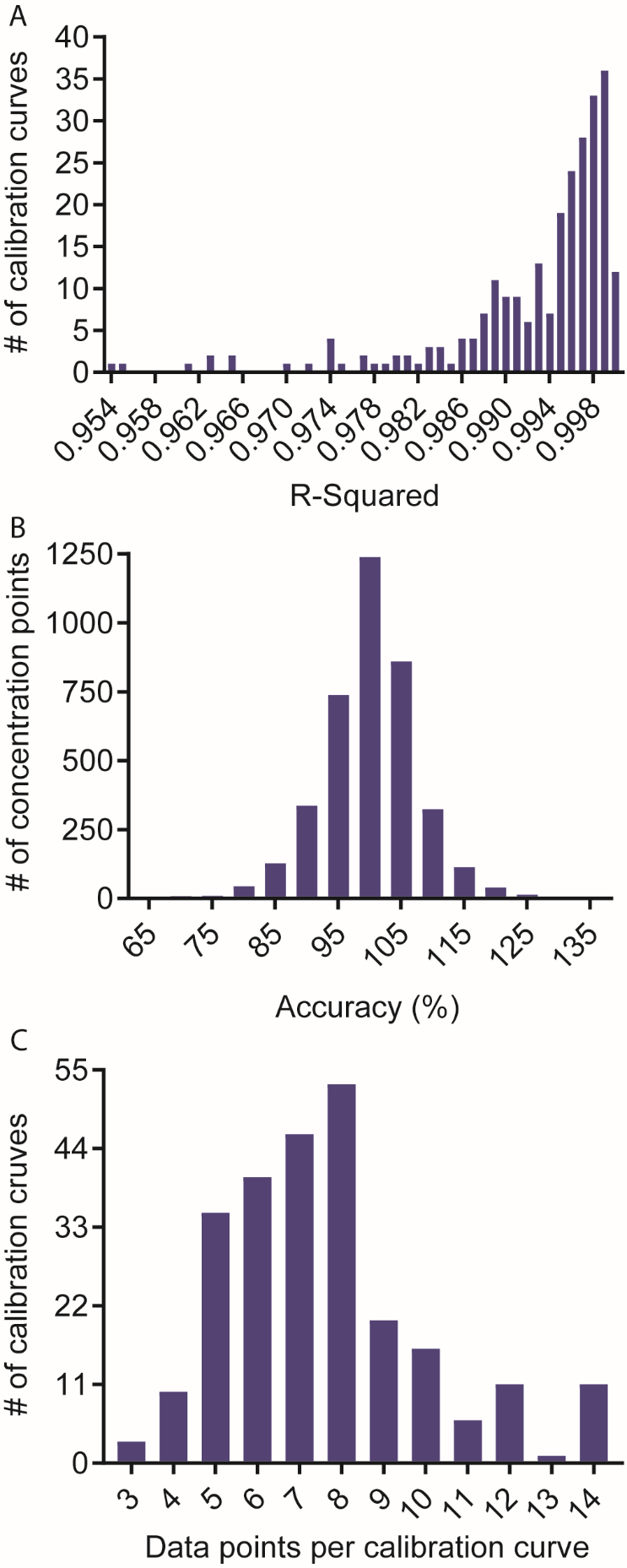


**supplemental Fig.S5. Analytical performance of calibration curves**. Endogenous protein concentration measurements were performed using external calibration curves, generated in a surrogate DBS matrix using SIS normalizers and varying the concentration of NAT. The performance of the calibration curves was established by maintaining high R-squared values, accuracy and number of data points used to generate the calibration curve. Data is representative of 252 peptides targeting 215 proteins **A**. R-squared values are presented in a frequency histogram with the number of MRM assays plotted on the y-axis and respective R-Squared values plotted on the x-axis. The median R-Squared value was 0.9959 and maximum and minimum values of 1 and 0.9541, respectively. **B**. Likewise, the accuracy of the calibration curves for measuring the NAT peptide used to generate data points is plotted as a frequency histogram of the number of concertation points measured across all calibration curves, which are plotted on the y-axis and the respective accuracies plotted on the x-axis. The median accuracy was 100.4% and maximum and minimums of 139.1% and 66.2%, respectively. **C**. The number of data points used to generate calibration curves is also plotted as a frequency histogram with the number of calibration curves plotted on the y-axis and the respective data points per calibration curve plotted on the x-axis. The median number of data points per calibration curve was 7, with a maximum and minimums of 14 and 3 data points, respectively.
